# Neddylation inhibition sensitises renal medullary carcinoma tumours to platinum chemotherapy

**DOI:** 10.1002/ctm2.1267

**Published:** 2023-05-25

**Authors:** Daniel D. Shapiro, Niki Millward Zacharias, Durga N. Tripathi, Menuka Karki, Jean‐Philippe Bertocchio, Melinda Soeung, Rong He, Mary E. Westerman, Jianjun Gao, Priya Rao, Truong N. A. Lam, Eric Jonasch, Luigi Perelli, Emily H. Cheng, Alessandro Carugo, Timothy P. Heffernan, Cheryl L. Walker, Giannicola Genovese, Nizar M. Tannir, Jose A. Karam, Pavlos Msaouel

**Affiliations:** ^1^ Department of Urology University of Wisconsin School of Medicine and Public Health Madison Wisconsin USA; ^2^ Division of Urology William S. Middleton Memorial Veterans Hospital Madison Wisconsin USA; ^3^ Department of Urology The University of Texas MD Anderson Cancer Center Houston Texas USA; ^4^ Center for Precision Environmental Health Baylor College of Medicine Houston Texas USA; ^5^ Department of Genitourinary Medical Oncology The University of Texas MD Anderson Cancer Center Houston Texas USA; ^6^ Department of Pathology The University of Texas MD Anderson Cancer Center Houston Texas USA; ^7^ Human Oncology & Pathogenesis Program and Department of Pathology Memorial Sloan Kettering Cancer Institute New York New York USA; ^8^ Institute for Applied Cancer Science The University of Texas MD Anderson Cancer Center Houston Texas USA; ^9^ Translational Research to Advance Therapeutics and Innovation in Oncology The University of Texas MD Anderson Cancer Center Houston Texas USA; ^10^ Department of Oncology IRBM Spa Rome Italy; ^11^ Department of Genomic Medicine The University of Texas MD Anderson Cancer Center Houston Texas USA; ^12^ David H. Koch Center for Applied Research of Genitourinary Cancers The University of Texas MD Anderson Cancer Center Houston Texas USA; ^13^ Department of Translational Molecular Pathology The University of Texas MD Anderson Cancer Center Houston Texas USA

**Keywords:** chemotherapy, neddylation, pevonedistat, renal medullary carcinoma

## Abstract

**Background:**

Renal medullary carcinoma (RMC) is a highly aggressive cancer in need of new therapeutic strategies. The neddylation pathway can protect cells from DNA damage induced by the platinum‐based chemotherapy used in RMC. We investigated if neddylation inhibition with pevonedistat will synergistically enhance antitumour effects of platinum‐based chemotherapy in RMC.

**Methods:**

We evaluated the IC_50_ concentrations of the neddylation‐activating enzyme inhibitor pevonedistat in vitro in RMC cell lines. Bliss synergy scores were calculated using growth inhibition assays following treatment with varying concentrations of pevonedistat and carboplatin. Protein expression was assessed by western blot and immunofluorescence assays. The efficacy of pevonedistat alone or in combination with platinum‐based chemotherapy was evaluated in vivo in platinum‐naïve and platinum‐experienced patient‐derived xenograft (PDX) models of RMC.

**Results:**

The RMC cell lines demonstrated IC_50_ concentrations of pevonedistat below the maximum tolerated dose in humans. When combined with carboplatin, pevonedistat demonstrated a significant in vitro synergistic effect. Treatment with carboplatin alone increased nuclear ERCC1 levels used to repair the interstrand crosslinks induced by platinum salts. Conversely, the addition of pevonedistat to carboplatin led to p53 upregulation resulting in FANCD2 suppression and reduced nuclear ERCC1 levels. The addition of pevonedistat to platinum‐based chemotherapy significantly inhibited tumour growth in both platinum‐naïve and platinum‐experienced PDX models of RMC (*p* < .01).

**Conclusions:**

Our results suggest that pevonedistat synergises with carboplatin to inhibit RMC cell and tumour growth through inhibition of DNA damage repair. These findings support the development of a clinical trial combining pevonedistat with platinum‐based chemotherapy for RMC.

## Graphical abstract

Critical points:

- Inhibition of neddylation by pevonedistat enhances the in vitro and in vivo antitumor effects of platinum‐based chemotherapy in renal medullary carcinoma (RMC) cell lines and patient derived xenografts.
- Pevonedistat induces p53‐mediated suppression of FANCD2 resulting in reduction of ERCC1 recruitment at locations of DNA damage.
- Inhibition by pevonedistat of platinum‐induced DNA damage repair increases RMC cellular apoptosis.

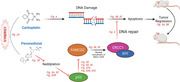

## 1 BACKGROUND

Renal medullary carcinoma (RMC) is a lethal malignancy arising from the renal medulla primarily in patients of African descent with sickle haemoglobinopathies at a median age of 28 years old.^1^, ^2^, ^3^ Current treatments used for other renal cell carcinomas such as tyrosine kinase inhibitors and immune checkpoint therapies have limited utility in RMC and patients are primarily managed with cytotoxic chemotherapy.^2^ Despite best available therapies, the median survival of patients with RMC is only 13 months; thus, there is a need to identify novel biologically informed treatment strategies.^1^, ^2^, ^4^, ^5^ Protein expression of the SMARCB1 tumour suppressor, a key component of the SWI/SNF ATP‐dependent chromatin remodelling complex, is lost in all RMC tumours mainly via inactivating deletions and translocations of the *SMARCB1* gene.^6^, ^7^, ^8^, ^9^ SMARCB1 loss upregulates c‐MYC leading to profound cellular proteotoxic and replication stress.^8^, ^10^, ^11^, ^12^, ^13^ This proteotoxic stress makes RMC tumours susceptible to agents such as proteasome inhibitors that deregulate the cellular proteostatic machinery.^8^, ^10^, ^11^, ^12^, ^13^ Furthermore, the replication stress induced by SMARCB1 loss confers a therapeutic vulnerability of RMC to agents that induce DNA damage, such as platinum salts.^8^, ^14^ Platinum‐based cytotoxic chemotherapy, such as the combination of carboplatin with paclitaxel, is currently the recommended frontline therapy for RMC but demonstrates an objective response in only 29% of patients.^1^, ^2^ Improving the response rate of RMC to platinum‐based regimens is therefore warranted.

Neddylation is a posttranslational modification that is integral to the ubiquitin–proteasome pathway and controls around 20% of ubiquitin‐mediated proteolysis.^15^ It consists of the covalent linkage of the small molecule NEDD8 to members of the cullin‐ring ligase (CRL) family, which are in turn responsible for substrate‐specific ubiquitination leading to protein degradation.^16^, ^17^ The first step in the neddylation pathway is the activation of NEDD8 by the NEDD8‐activating enzyme (NAE). Pevonedistat is a small molecule inhibitor of NAE that prevents the neddylation of CRLs.^17^, ^18^ Prior studies have demonstrated that inhibition of the neddylation pathway by pevonedistat aggravates both proteotoxic and replication stress.^19^, ^20^ The proteotoxic stress induced by SMARCB1 loss has accordingly been shown to confer sensitivity to pevonedistat in malignant rhabdoid tumour (MRT) models.^20^ Furthermore, inhibition of the neddylation pathway may sensitise cells to platinum chemotherapy by reducing the FANCD2‐mediated recruitment of the XPF–ERCC1 nucleotide excision repair (NER) complex to DNA interstrand cross‐links (ICL) induced by platinum salts.^21^ This potentially synergistic activity is supported by clinical data from a phase I trial where the addition of pevonedistat to carboplatin and paclitaxel yielded an objective response rate of 35%, including two complete responses (9%) in a patient with urothelial carcinoma and a patient with endometrial cancer who were previously refractory to platinum‐based chemotherapy.^22^ Of note, even though elevated ERCC1 levels are associated with resistance to platinum agents due to increased ICL repair,^23^ trial patients with high ERCC1 expression in their tumours derived the most clinical benefit from the addition of pevonedistat to carboplatin and paclitaxel.^22^

The efficacy of carboplatin + paclitaxel in RMC may be attenuated by the twofold higher ERCC1 RNA expression noted in RMC tissues compared to normal kidney.^8^ We therefore hypothesised that pevonedistat will enhance the efficacy of carboplatin in combination with paclitaxel in RMC tumours by inhibiting the FANCD2‐mediated recruitment of XPF–ERCC1 to DNA ICLs induced by carboplatin. We tested this hypothesis in vitro in RMC cell lines, and in vivo in two separate patient‐derived xenograft (PDX) models of RMC and found that neddylation inhibition can synergistically enhance the efficacy of platinum‐based chemotherapy paving the way for clinical trials in RMC.

## 2 METHODS

### 2.1 Cell lines

The RMC2C cell line, which originates from the primary nephrectomy sample of a 35‐year‐old African American man with sickle cell trait, was developed as previously detailed,^24^ and authenticated using short tandem repeat (STR) DNA profiling to compare with the original patient‐derived tissue.^25^ RMC2C was maintained at 37°C in minimum essential medium (MEM) supplemented with non‐essential amino acids from MEM, EGF (5 μg/mL), penicillin–streptomycin (100 U/mL) and 10% heat‐inactivated foetal bovine serum. The RMC219 cell line was derived from a PDX from the bone metastasis of a 39‐year‐old African American man with sickle cell trait who had received five cycles of gemcitabine and carboplatin for metastatic RMC as previously described.^26^ RMC219 was grown at 37°C in Ham's F‐12 medium with 1% essential amino acids, 1% sodium pyruvate and 1% L‐glutamine. All media were supplemented with 100 U/mL penicillin–streptomycin and 10% heat‐inactivated foetal bovine serum. Two more SMARCB1‐deficient cell lines, G401 and VA‐ES‐BJ, were acquired from the American Type Culture Collection (ATCC, Manassas, VA, USA). G‐401 is an MRT cell line, while VA‐ES‐BJ is an epithelioid sarcoma cell line. Mycoplasma monitoring for these cell lines was performed using ATCC's Universal Mycoplasma Detection Kit (Manassas, VA, USA).

### 2.2 Cell viability assays

To determine synergy between pevonedistat and carboplatin or pevonedistat and paclitaxel, we employed the Bliss independence model, which postulates that two drugs exert their effects independently through a stochastic process. The anticipated combined effect can be determined by considering the probability of independent events.^27^ Using the SynergyFinder application,^27^ we calculated Bliss summary synergy scores for dose–response matrix data using results from MTT assays. Each summary score was calculated using the combined results of quadruplicate MTT assays. Cells were exposed to the following drug combinations: pevonedistat (0–0.5 μM) and carboplatin (0–80 μM) or pevonedistat (0–1.0 μM) and paclitaxel (0–10 nM). Cells were treated for 48 h. For the in vitro synergy experiment, when monotherapy was being evaluated, the cells were treated with the experimental drug as well as a DMSO vehicle control. The summary synergy scores can be understood as the mean additional response resulting from the interaction between drugs. Two‐drug combinations that result in summary scores less than −10 indicate an antagonistic relationship. Scores ranging from −10 to 10 indicate that the drugs have additive effects, and scores greater than 10 suggest that the two drugs likely have a synergistic effect.^27^ All synergy experiments were performed in triplicate. The concentrations of chemotherapy drugs used in the in vitro experiments were selected to be lower than their corresponding peak plasma concentrations found in humans.^28^, ^29^, ^30^

### 2.3 Western blot analyses

Protein concentrations were determined using the Pierce BCA protein assay kit. Samples were combined with an equal volume of Laemmli Sample Buffer (Bio‐Rad) and heat‐denatured (100°C, 10 min) with β‐mercaptoethanol (Sigma–Aldrich). The samples were then loaded onto precast SDS/PAGE gels (Bio‐Rad), transferred to PVDF membranes, and incubated with specific primary antibodies overnight at 4°C. The next day, membranes were treated with secondary anti‐mouse or anti‐rabbit immunoglobulin G conjugated horseradish peroxidase (HRP) antibodies, and chemiluminescence was detected through film exposure. To minimise non‐specific signals, membranes were blocked with 3% bovine serum albumin (BSA) or Odyssey blocking buffer (Licor). The primary antibodies used included: mouse monoclonal anti‐SMARCB1 antibody clone 2C2 (Sigma–Aldrich; SAB4200202), rabbit polyclonal anti‐CUL1 (Cell Signaling Technology; 4995), rabbit polyclonal anti‐PARP (Cell Signaling Technology; 9542), goat polyclonal anti‐ATR (Santa Cruz Biotechnology; sc‐1887), rabbit polyclonal anti‐phospho‐ATR at serine 428 (Cell Signaling Technology; 2853), mouse monoclonal anti‐TP53 (Santa Cruz Biotechnology; sc‐126), rabbit polyclonal anti‐phospho‐TP53 at serine 15 (Cell Signaling Technology; 9284), mouse monoclonal anti‐actin (Santa Cruz Biotechnology; sc‐47778), mouse monoclonal anti‐FANCD2 (Santa Cruz Biotechnology; sc‐20022) and mouse monoclonal anti‐ERCC1 (Santa Cruz Biotechnology; sc‐17809).

Quantification of protein expression levels was carried out through densitometric analysis using ImageQuant TL software, with data normalised to β‐actin. For the western blots of phosphorylated proteins (ATM, ATR and TP53), data were normalised using total protein and presented as the ratio of phosphorylated to total protein. All samples were conducted in triplicate.

### 2.4 Apoptosis assay using flow cytometry

The measurement and quantification of apoptosis in RMC2C cells treated with a drug or combination of drugs was conducted using an Annexin V‐FITC/propidium iodide (PI) staining kit (BD Bioscience). RMC2C cells were first seeded in a six‐well plate and treated with either DMSO (Control), pevonedistat 0.2 μM, carboplatin 20 μM or pevonedistat with carboplatin for 48 h. The cells were then washed in phosphate‐buffered saline (PBS) and harvested by trypsinisation. These harvested cells were washed once with ice‐cold PBS and then with 500 μL of 1× binding buffer. Cells were incubated with FITC Annexin V in 1× binding buffer containing PI in dark at 4°C for 15 min. Following removal of stain, cells were resuspended in 1× binding buffer and then examined using BD FACS Canto II flow cytometry (BD Bioscience, NJ, USA). The obtained flow cytometry data was analysed and graphed using FlowJo_v10.8.1 software.

### 2.5 CRISPR/Cas9 knockout

*TP53* knockout was accomplished using lentivirus produced in HEK‐293FT cells with pLentiCRISPR v2 plasmids containing TP53‐targeting gRNA sequences, acquired from Genscript (Piscataway, NJ, USA). Lentiviral packaging was carried out using psPAX2, a gift from Didier Trono (Addgene plasmid #12260; http://n2t.net/addgene:12260; RRID:Addgene_12260), and lentiviral envelope expression was achieved using pMD2.G, also a gift from Didier Trono (Addgene plasmid # 12259; http://n2t.net/addgene:12259; RRID:Addgene_12259). Lentiviral‐transduced cells were selected using puromycin. As a negative control, cells transduced by lentivirus generated with a pLentiCRISPR v2 plasmid containing non‐targeting control gRNA (BRDN0001145885) were used,^31^ a gift from John Doench & David Root (Addgene plasmid #80196; http://n2t.net/addgene:80196; RRID:Addgene_80196). All plasmid vectors were propagated using the *Escherichia coli* strain DH5α (Invitrogen; Cat#18265017).

gRNA target sequences used for human *TP53* knockout:

- CCGGTTCATGCCGCCCATGC
- CGCTATCTGAGCAGCGCTCA

Non‐targeting control gRNA sequence:

- GGGACGCGAAAGAAACCAGT

### 2.6 Small interfering RNA knockdown

*TP53* knockdown was accomplished using siGENOME Human TP53 small interfering RNA (siRNA) SMARTpool at a 10 nM concentration or a sham control provided by Horizon Discovery (Lafayette, CO, USA). In the siRNA knockdown western blot experiments, cells were initially treated with siRNA targeting p53 or the sham control for 48 h before starting drug treatments with pevonedistat and carboplatin for another 48 h.

### 2.7 Immunofluorescence analysis

RMC2C cells were placed onto six‐well culture dishes with coverslips and treated with compounds (control, pevonedistat 0.2 μM, carboplatin 20 μM or pevonedistat 0.2 μM + carboplatin 20 μM) for 48 h. Subsequently, the cells were fixed with 4% paraformaldehyde (PFA) in PBS for 15 min at room temperature and permeabilised with 0.5% Triton X‐100 in PBS for 15 min. The cells were then blocked using 3.75% BSA in PBS for 1 h. After blocking, primary mouse monoclonal anti‐ERCC1 (Santa Cruz Biotechnology; sc‐17809) and rabbit monoclonal phospho‐histone H2A.X (Ser139, 20E3) antibodies for detecting γH2AX (Cell Signaling; 9718) were added in a 3.75% BSA solution at ratios of 1:100 and 1:250, respectively, and incubated overnight at 4°C. The cells were then washed three times for 10 min each with PBS and incubated with mouse Alexa Fluor 546 and/or rabbit Alexa Fluor 488‐labelled secondary antibodies (Invitrogen, Eugene, OR) at a 1:2000 dilution at room temperature for 1 h. After washing the cells three times with PBS for 10 min each, they were postfixed with 4% PFA for 10 min to stabilise the signal. Cells were counterstained with DAPI (Invitrogen; diluted 1:4000) for 10 min to visualise DNA. Coverslips were mounted using Prolong™ Gold Antifade Mountant (Invitrogen, P36930). Fixed cells were imaged with a CFI Plan Apochromat Lambda 60× oil, 1.4 numerical aperture (NA) objective, and DSQi2 camera on a Nikon Eclipse Ti2‐E inverted research microscope (Nikon Instruments, Melville, NY, USA) equipped for standard phase contrast and epifluorescence microscopy as well as deconvolution. All the images were taken at fixed exposure time for the red channel (ERCC1) and blue channel (DAPI), and signal intensity was measured using Nikon NIS‐Element software. Approximately 100 cells were taken into consideration for quantification from each group. Statistical analysis was carried out using Prism software.

For acquisition of the high‐resolution images shown in Figure 4, an HC PL APO 100×/1.40 CS2 laser scanning confocal microscope (Leica Sp8) with spectral emission detection on an inverted stand, fully motorised stage, fast z movement (Leica Super Z Galvo stage) with resolution up to ∼20 nm was used that allowed observation of greater details of subcellular structures. All the acquired images were then processed using Leica's LASX software. Additionally, for the analysis of foci, images were also acquired using inverted Leica DMi8 widefield fluorescence microscope equipped with epifluorescence lamp and EM‐CCD camera with HC PL APO 63× Oil, NA 1.4. All the images were processed using Leica's LASX software and were further analysed for foci using Fiji (ImageJ) software.

### 2.8 Quantitation of nuclear foci of  γH2AX and ERCC1

To quantify γH2AX and ERCC1 foci in the nuclei, Gaussian filter (included within the ‘Filter’) of the Fiji (ImageJ) software was used to reduce the unnecessary noise and extract the quantifiable smoothened pixel features for foci count. Following the application of Gaussian filters, the foci count was generated using ‘Analyse Particle’, where size of 5‐infinity pixel units was considered for any foci to be counted. All the foci counted were graphed and analysed using GraphPad Prism software.

### 2.9 Colocalisation assay

For the colocalisation assay, acquired immunofluorescence images were analysed by Fiji (ImageJ) software using Bioimaging and Optics Platform (BIOP) version of Just Another Colocalisation Plugin (JACoP). Spearman's rank correlation coefficient values were obtained after applying the plugin, graphed and analysed to determine the colocalisation or correlation between proteins using GraphPad Prism software.

### 2.10 Patient‐derived xenograft models

Animal studies, including housing, care, euthanasia methods and experimental protocols, were approved by the University of Texas MD Anderson Cancer Center Animal Care and Use Committee, adhering to relevant guidelines. Two xenograft models were developed from patient‐derived tumours. The first (RMC2X) was derived from the same RMC primary tumour specimen as the cell line RMC2C using previously described methodology.^32^ A primary tumour sample was implanted orthotopically and subcutaneously and allowed to grow for three serial passages within SCID mice (Figure S1A). After the third passage, the xenograft tumour was compared to the primary tumour using STR DNA profiling to confirm the xenograft tumour maintained patient‐derived tumour tissue using the Promega 16 High Sensitivity STR Kit (catalogue #DC2100)^25^ (Supporting Information S1). A second PDX model (RMC32X) was developed in a similar fashion. RMC32X was derived from the nephrectomy specimen of a black male patient with sickle cell trait who had received platinum therapy prior to nephrectomy; thus, the PDX served as a model of a platinum‐experienced tumour. Both primary tumours from which the two PDX models were derived have been previously molecularly characterised.^8^

### 2.11 Xenograft studies

Tumour fragments (30–40 mg) were implanted subcutaneously in 6‐ to 8‐week‐old male and female CB17/lcr‐*Prkdc^scid^*/lcrlcoCrl mice (Charles River). Tumours were allowed to grow to approximately 200 mm^3^ prior to the initiation of drug therapy. Tumours were measured using callipers, and volume (mm^3^) was calculated using the formula (length × width^2^)/2. Once tumours were of adequate volume, the mice were exposed to one of four treatments, vehicle control (5% DMSO in PBS), pevonedistat (30 mg/kg dissolved in 5% DMSO [Selleckchem]), carboplatin (80 mg/kg) with paclitaxel (20 mg/kg), or triple therapy combining pevonedistat, carboplatin and paclitaxel. For in vivo experiments, the concentrations of chemotherapy drugs used were selected to be lower than their respective peak plasma concentrations in humans.^28^, ^29^, ^30^ Carboplatin and paclitaxel were purchased from the MD Anderson Investigational Pharmacy and diluted with PBS. All treatments were injected intraperitoneally. The vehicle control and pevonedistat were given daily for 5 and 2 days off for each 7‐day cycle. Carboplatin and paclitaxel were given once per 7‐day cycle. Tumours were measured, and mice were typically weighed on days 1, 3 and 5 of each cycle. Investigators making measurements were blinded to treatment group. To determine whether a synergistic tumour inhibitor effect existed when combining pevonedistat with carboplatin and paclitaxel in vivo, we performed a statistical determination of synergy based on the Bliss definition of drug independence.^33^ Drug independence is based on the equation $\alpha +\beta_{1}t+\alpha +\beta_{2}t-2\left(\alpha +\beta_{0}t\right)=\alpha +\beta_{3}t-\alpha -\beta_{0}t$, where $\beta_{0},\;\beta_{1},\;\beta_{2}\;\;\mathrm{and}\;\beta_{3}\;$are the tumour growth rates for the control, pevonedistat, carboplatin with paclitaxel and triple therapy (pevonedistat with carboplatin and paclitaxel) treatment groups, respectively. To facilitate these estimations, all tumours were excised after the same amount of time. The RMC2X tumours grew more rapidly than the RMC32X tumours; thus, the experiment of RMC2X was ended on day 19 and RMC32X was ended on day 26. After cancellation of time *t*, the hypothesis of Bliss independence can be rewritten in terms of rate coefficients $H_{0}=\beta_{1}+\beta_{2}-\beta_{3}-\beta_{0}=0$. We considered there to be synergy if $\beta_{1}+\beta_{2}-\beta_{3}-\beta_{0}>0$ was statistically supported by a low *p‐*value computed from estimates of the slope of the plotted relative tumour volumes and their variances.^33^ Synergy would not be considered present if $\beta_{1}+\beta_{2}-\beta_{3}-\beta_{0}\leq 0$.

### 2.12 Immunohistochemistry

To determine the effects of the various therapies on PDX tumour cellular protein expression, RMC32X tumours from each therapy arm were excised simultaneously at the end of the experiment (day 26) to control for the amount of time the tumours were exposed to each therapy. The excised PDX tumours were formalin fixed and paraffin embedded (FFPE). FFPE tissue sections were used for FANCD2 immunohistochemical staining. Slides with tissue sections were baked in an oven at 60°C for 1 h. Slides were then dewaxed using xylene then rehydrated using an ethanol gradient of 100% then 95% ethanol, anhydrous. To perform antigen unmasking, slides were then placed in a plastic coplin jar and filled to the top with AR6 buffer from Akoya Biosciences (AR6001KIT). The entire jar and slides underwent microwave treatment of 55 s at 100% power and then 15 min at 20% power using a Panasonic Inverter microwave. Slides were left at room temperature afterwards to cool. A humidified chamber was prepared for all the subsequent incubation steps. To block endogenous peroxidases and reduce background staining, slides were incubated in 3% hydrogen peroxide for 10 min. Secondary antibody host was blocked by incubating the slides with 2.5% normal goat serum (Vector Laboratories, S‐1012‐50) for 30 min at room temperature. The slides were then incubated overnight at 4°C in anti‐FANCD2 antibody (Abcam, ab108928) prepared in SignalStain antibody diluent (Cell Signaling, 8112S) at a concentration of 1:50. After the overnight primary antibody incubation, the slides were incubated in SignalStain Boost IHC Detection, HRP rabbit (Cell Signaling, 8114) for 30 min at room temperature. Immunohistochemical staining was performed using the ImmPact 3,3′‐diaminobenzidine (DAB) peroxidase (HRP) Substrate (Vector Laboratories, SK‐4105). Each slide was incubated with 200 μL of the substrate for up to 10 min (until dark staining could be detected). Slides were then counterstained with Harris haematoxylin, dehydrated with 95% and 100% ethanol, anhydrous and then xylene. Finally, slides were mounted with coverslips using Cytoseal mounting medium.

### 2.13 FANCD2 immunohistochemistry imaging and quantification

Images are first taken with the Vectra 3 automated quantitative pathology imaging system. Five images were captured for each of the four categories of tissues (control, pevonedistat, carboplatin + paclitaxel and pevonedistat + carboplatin + paclitaxel). The images were then processed for quantification of the FANCD2 immunohistochemical staining using the Inform 2.5 software from Akoya Biosciences. Images are initially prepared based on a spectral library that detects DAB (FANCD2) and haematoxylin (nuclei). Based on DAB and haematoxylin stains, the inform software then performs cell segmentation to determine nuclear and cytoplasm compartments for each cell in the image. In the last step, FANCD2 staining is scored by assigning the nuclei compartment (for FANCD2) with the DAB component. To accurately score FANCD2 staining, a threshold is set for DAB positivity which produces a scoring map that matches the staining in the original images. Once a threshold has been set, it is applied to all images, all images are scored, and a percent positivity is given for each image. The percent positivity represents the percent of cells in the given image that are positive for DAB staining, which in this case represents the percentage of FANCD2‐positive cells. Fisher's exact tests were used to test for differences in the proportions of FANCD2 positivity between individual experimental arms. *p*‐Values less than .05 were considered statistically significant.

### 2.14 Statistical analysis

Unless otherwise mentioned, all experiments were conducted in triplicate, and statistical significance was determined using either an unpaired *t*‐test or Fisher's exact test with GraphPad Prism software. A *p*‐value of less than .05 was deemed statistically significant.

## 3 RESULTS

### 3.1 SMARCB1‐deficient cell lines are sensitive to pevonedistat

**FIGURE 1:**
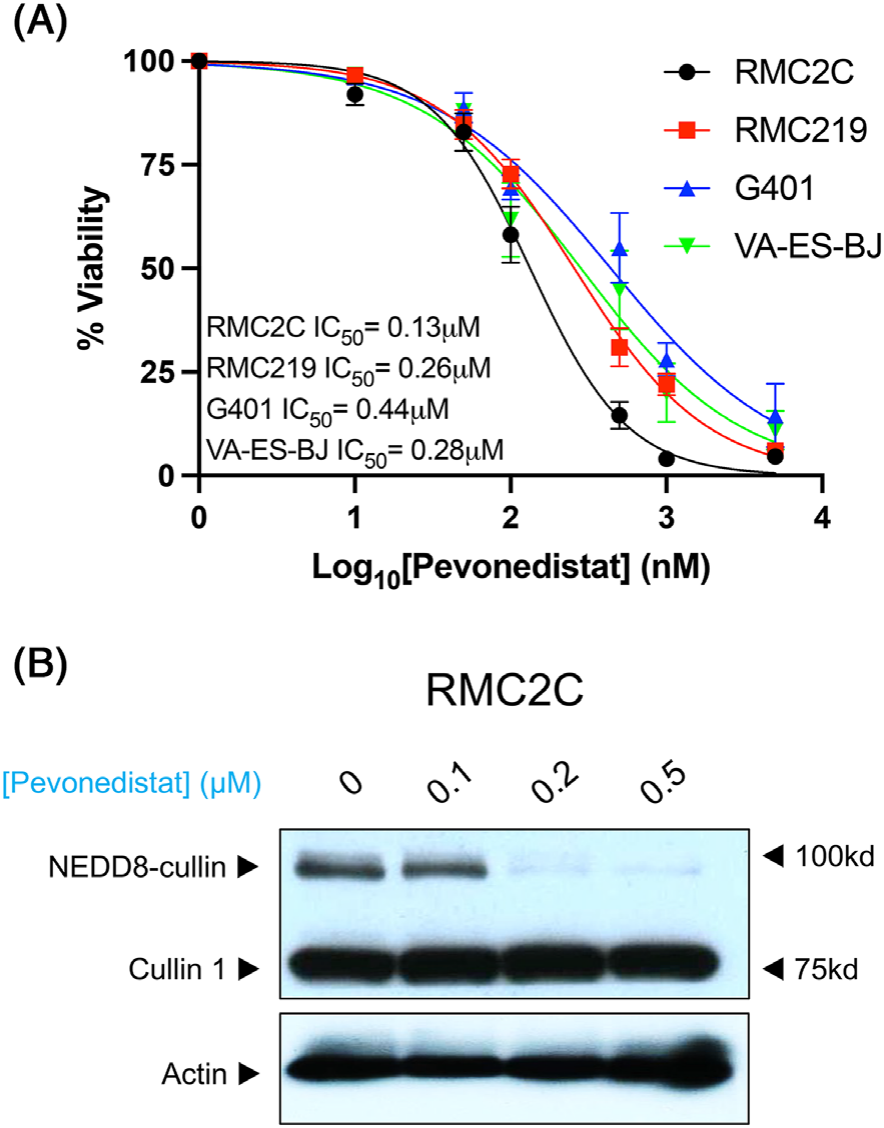
SMARCB1‐deficient cell lines are sensitive to pevonedistat. (A) Dose response curves for four SMARCB1‐deficient cell lines including two renal medullary carcinoma cell lines (RMC2C, RMC219), a malignant rhabdoid tumor cell line (G401) and an epithelioid sarcoma cell line (VA‐ES‐BJ) after exposure to varying concentrations of pevonedistat. IC_50_ concentrations are listed for each cell line and error bars indicate standard error of the mean (SEM) for quadruplicate measurements. (B) Western blot analysis of Cullin 1 following exposure to increasing concentrations of pevonedistat after 48 hours of treatment. Upper bands demonstrate reduction of the neddylated Cullin 1 with increasing dose of pevonedistat. RMC, renal medullary carcinoma.

To test the ability of pevonedistat to inhibit RMC and other SMARCB1‐deficient cell lines, we performed MTT assays on two RMC cell lines (RMC2C and RMC219) and in G‐401 (MRT) and VA‐ES‐BJ (epithelioid sarcoma) cell lines. Pevonedistat demonstrated efficacy at inhibiting all SMARCB1‐deficient cell lines, with the RMC cell lines achieving IC_50_ concentrations of 0.13 μM for RMC2C and 0.26 μM for RMC219 (Figure 1A). These concentrations are in concordance with currently estimated maximum tolerated doses in human clinical studies.^34^ Western blots demonstrated that pevonedistat achieved on‐target effects by inhibiting the neddylation of cullin 1 ring ligase in RMC2C cells (Figure 1B). Given that prior studies have demonstrated that SMARCB1 loss leads to profound replication and proteotoxic stress,^8^, ^10^, ^11^ and that neddylation inhibition can further promote these toxicities within cells, we hypothesised that the addition of pevonedistat to cytotoxic chemotherapy such as carboplatin could synergistically lead to RMC cell death and tumour growth inhibition.

### 3.2 RMC cell lines are synergistically sensitive to pevonedistat in combination with carboplatin

**FIGURE 2:**
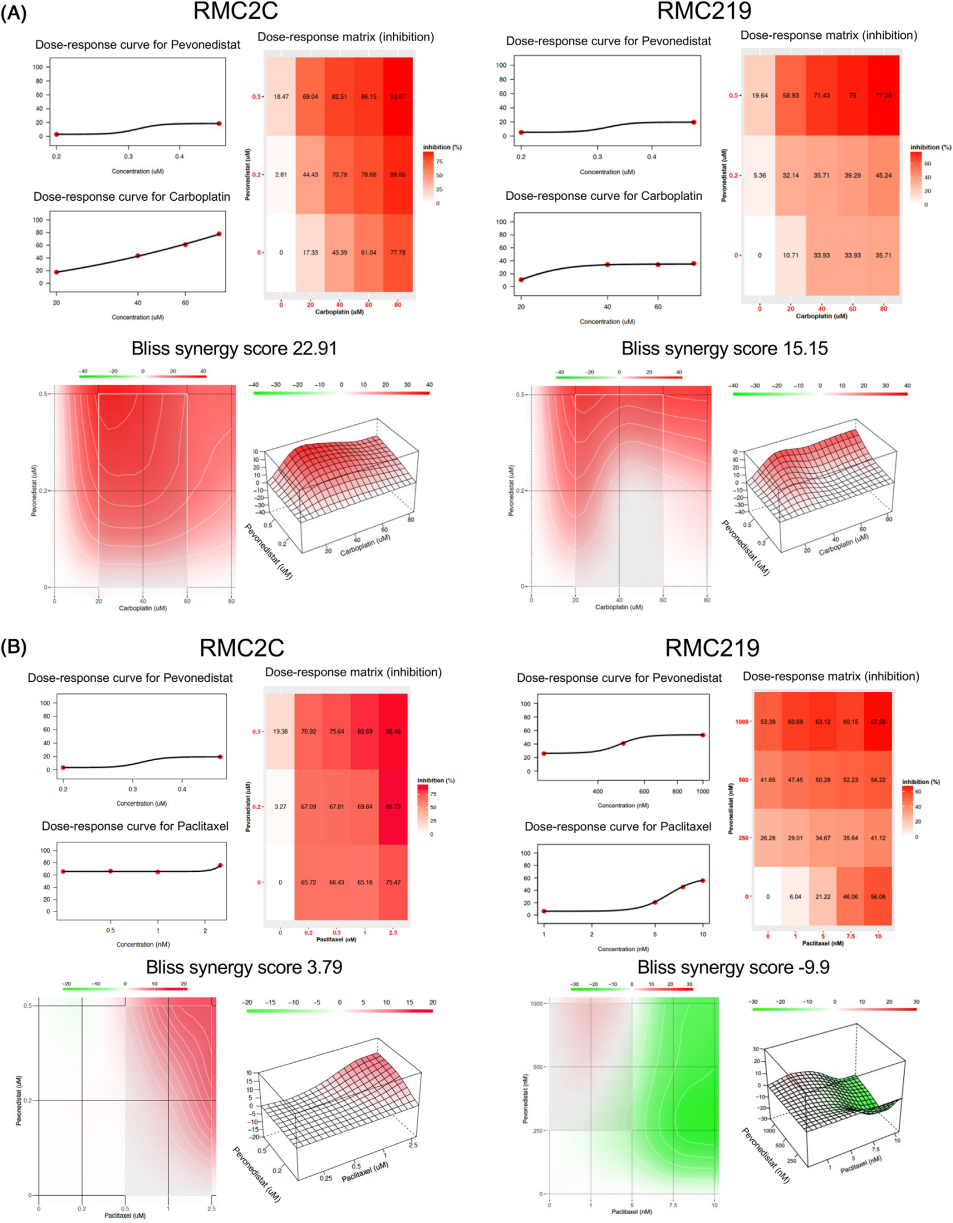
Pevonedistat synergises with carboplatin in renal medullary carcinoma (RMC) cell lines. (A and B) Dose–response data for the pairwise combination of pevonedistat (0–0.5 μM) with carboplatin (0–80 μM) (A) and pevonedistat (0–1.0 μM) with paclitaxel (0–10 nM) (B) in RMC2C and RMC219 cell lines. Dose–response curves are shown for each individual drug. The dose–response matrices demonstrate the percent of cellular inhibition of the treated cells compared to control cells at various concentrations of the drug combinations. Synergy scores are listed above the 2D and 3D synergy maps and calculated according to the Bliss method using quadruplicate MTT assay results. Scores >10 indicate a synergistic combination, scores −10 to 10 indicate additive effects, and scores less than −10 indicate antagonism. The 2D and 3D synergy maps highlight synergistic and antagonistic dose regions in red and green colours, respectively. These maps help to highlight the most synergistic areas. All synergy experiments were performed in triplicate.

We next sought to determine whether there is a synergistic effect of pevonedistat with either carboplatin or paclitaxel chemotherapy, with our hypothesis being that pevonedistat acts most synergistically with carboplatin. MTT assays were performed using various concentrations of pevonedistat (0–0.5 μM) and carboplatin (0–80 μM). Dose–response matrices were constructed using the SynergyFinder application,^27^ and Bliss synergy plots were constructed for RMC2C and RMC219 (Figure 2A). Both RMC2C and RMC219 demonstrated a synergistic effect between pevonedistat and carboplatin, yielding Bliss synergy scores of 22.91 for RMC2C and 15.15 for RMC219. Scores greater than 10 are considered to represent a synergistic effect between the two drugs.^27^ Because paclitaxel is frequently combined with carboplatin in the clinical setting, we further sought to evaluate if there is a synergistic effect between paclitaxel and pevonedistat. Unlike carboplatin, combining pevonedistat with paclitaxel only resulted in additive antitumour cell effects, yielding a Bliss synergy score of 3.79 in RMC2C and −9.9 in RMC219 (Figure 2B). Synergy scores between −10 and 10 are indicative of additive, but not synergistic, effects between two drugs.^27^ Overall, these results demonstrate a strong synergistic effect between carboplatin and pevonedistat in vitro and additive effects when pevonedistat is combined with paclitaxel.

### 3.3 The addition of pevonedistat to carboplatin suppresses the nucleotide excision repair pathway

**FIGURE 3:**
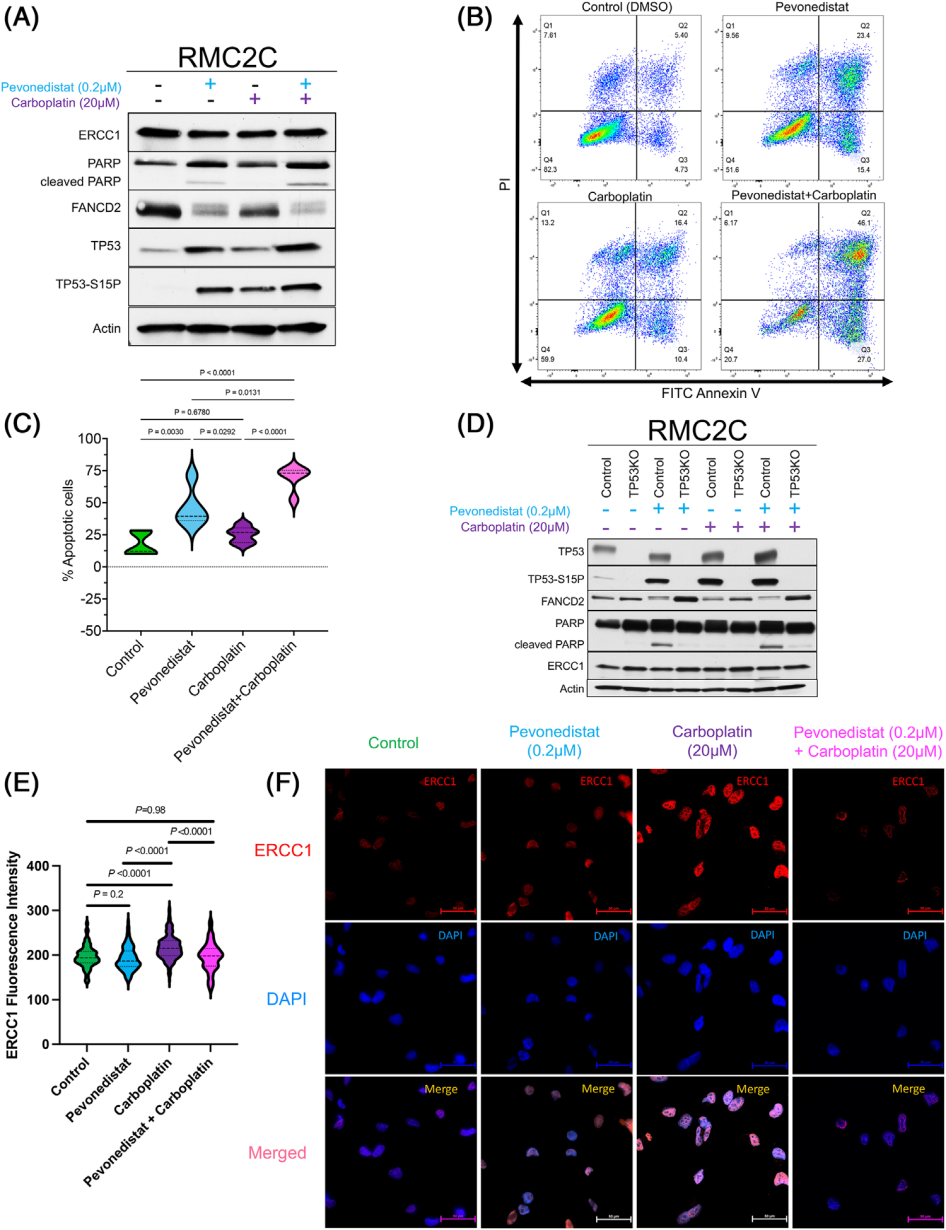
Pevonedistat inhibits activation of the nucleotide excision repair (NER) pathway. (A) Western blot analysis of components of the NER pathway. RMC2C cells were exposed to pevonedistat (0.2 μM) and carboplatin (20 μM) either individually or combined for 48 h and then western blots were performed for individual NER components. (B and C) Flow cytometric analysis of Annexin V‐FITC/propidium iodide (PI)‐stained control and drug treated RMC2C cells. (B) Representative images showing typical quadrant analysis of RMC2C cells with each quadrant representing the proportion (%) of cells with typical morphologies: Q1 represents necrotic cells, Q2 represents late apoptotic cells, Q3 represents early apoptotic cells and Q4 represents the viable cells. (C) Violin plot showing the percent of apoptotic cells by quantitative analysis using the values from (B). (D) TP53 CRISPR knockout RMC2C cells were created and then exposed to vehicle control, pevonedistat, carboplatin or pevonedistat combined with carboplatin for 48 h at which point western blotting was performed for the indicated proteins. (E and F) Immunofluorescence imaging of RMC2C cells immunolabelled for DAPI (blue) or ERCC1 (red) after treatment with DMSO, pevonedistat (0.2 μM), carboplatin (20 μM) or pevonedistat combined with carboplatin for 48 h. Violin plot in (E) demonstrates the ERCC1 fluorescent signal intensity for each treatment group after quantifying approximately 100 cells. Within the violin plot, dashed lines represent the median and dotted lines represent the upper and lower quartiles. Representative immunofluorescence images for each treatment group are shown in (F). Scale bars, 50 μm. *p*‐Values were generated using unpaired *t*‐test. RMC, renal medullary carcinoma.

To evaluate the mechanism behind the synergistic effect of pevonedistat and carboplatin, we exposed RMC2C cells to pevonedistat alone, carboplatin alone and pevonedistat combined with carboplatin for 48 h and determined the expression of proteins within the NER pathway, since this pathway is activated in response to carboplatin‐induced ICLs. We found that cells treated with pevonedistat resulted in a reduction in FANCD2 expression compared to control (*p* < .0001) and carboplatin treated cells (*p* < .0001) (Figures 3A and S2A). When cells were treated with both pevonedistat and carboplatin, there was further reduction in FANCD2 expression compared to cells treated with pevonedistat alone (*p* = .0004) (Figures 3A and S2A). Cells treated with pevonedistat and carboplatin demonstrated increased TP53 expression compared to control cells (*p* < .0001) and cells treated with either pevonedistat alone (*p* < .0001) or carboplatin alone (*p* < .0001) (Figures 3A and S2B). We noted that cells treated with pevonedistat or pevonedistat with carboplatin resulted in increased cleaved PARP, a marker of apoptosis (Figure 3A). Flow cytometric analysis of Annexin V‐FITC/PI‐stained RMC2C cells confirmed this finding and demonstrated that the combination of pevonedistat with carboplatin resulted in the highest number of apoptotic cells compared to control cells and cells treated with either pevonedistat or carboplatin monotherapy (Figure 3B,C).

Increased expression and activation of TP53 is known to lead to downregulation of FANCD2, a critical component of the NER pathway.^35^ We accordingly found that pevonedistat and carboplatin treatment increased the phosphorylation of TP53 at serine 15, a marker specific to DNA damage repair and not to other stimuli such as hyperproliferation^36^ (Figure 3A,D). Neddylation is also known to be an inhibitor of TP53 expression.^37^, ^38^ We thus hypothesised that by inhibiting the neddylation pathway, pevonedistat causes increased TP53 expression and subsequent FANCD2 suppression. To test this hypothesis, we performed a CRISPR knockout of *TP53* in RMC2C cell lines (Figure 3D). Compared with control cells, RMC2C *TP53* knockout cells resulted in effective TP53 suppression. As expected, pevonedistat exposure again caused a reduction in FANCD2 expression. However, this effect was not observed in *TP53* knockout cells. The same pattern was observed after treatment with both pevonedistat and carboplatin (Figure 3D). We further validated these findings by performing siRNA knockdown of TP53 in RMC2C cells and quantifying the FANCD2 expression (Figure S3A–C). We again found a significant increase in FANCD2 expression after TP53 knockdown with siRNA even when cells were exposed to pevonedistat and carboplatin (*p* = .002) (Figure S3C). These data support the hypothesis that pevonedistat results in increased TP53 expression, particularly the phosphorylated TP53–S15P, which is activated in response to DNA damage repair, and subsequent inhibition of the NER pathway mediated by FANCD2.

FANCD2 is a component of the Fanconi anaemia (FA) complex, which is known to promote the loading of the ERCC1/XPF complex at ICLs.^39^ While protein expression of ERCC1 did not significantly differ in our western blot assays (Figure 3A,D), we investigated for differences in the nuclear levels of ERCC1 in RMC2C cells after exposure to pevonedistat, carboplatin or pevonedistat combined with carboplatin. Using immunofluorescence assays, we found a significant increase in nuclear ERCC1 when exposed to carboplatin alone compared to control (*p <* .0001) and compared to pevonedistat alone (*p* < .0001). However, compared to carboplatin alone, the ERCC1 nuclear fluorescence was significantly attenuated (*p* < .0001) when pevonedistat was added to carboplatin (Figure 3C,D). In fact, the combination of pevonedistat with carboplatin reduced the ERCC1 signal to a level similar to that in control cells (*p =* .98). This again supports that pevonedistat mediates the suppression of FANCD2 and subsequent inhibition of the NER pathway.

**FIGURE 4:**
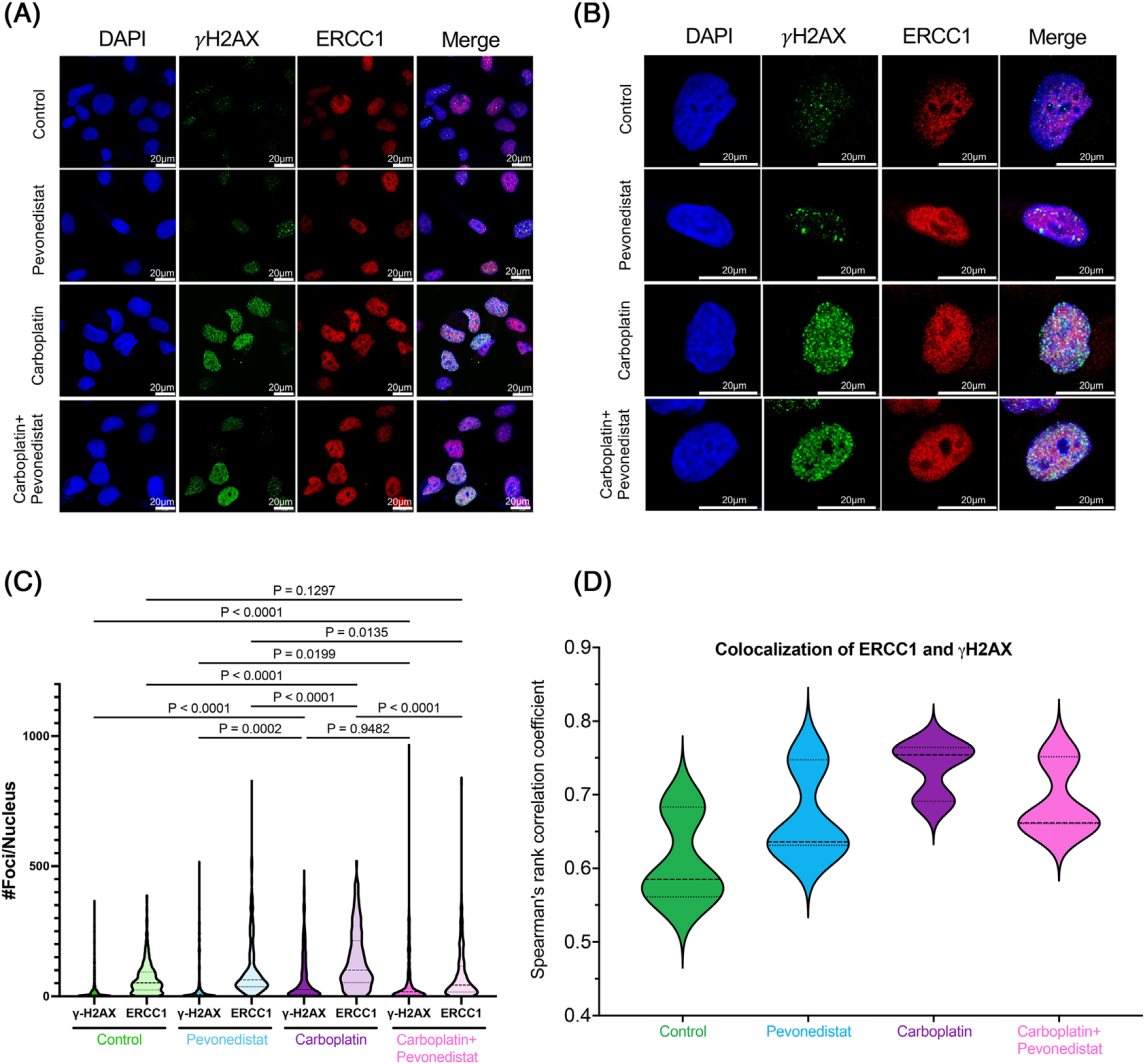
ERCC1 foci formation at sites of DNA damage. (A and B) Following 48 h of drug treatment, cells were fixed and immunostained for ERCC1 and γH2AX (a marker of DNA double strand breaks). Nuclei were stained with DAPI. Scale bar is 20 μm. (C) Cells treated with carboplatin alone showed increased γH2AX foci compared to either control or pevonedistat treated cells. When carboplatin was combined with pevonedistat, there was a significant decrease in ERCC1 foci compared to carboplatin monotherapy. (D) Colocalisation of ERCC1 and γH2AX foci was determined using Spearman's rank correlation coefficient. Significant colocalisation of proteins was considered if the median correlation was >0.5.

Additionally, we quantified the degree of ERCC1 foci formation using immunofluorescence. After 48 h of treatment, we evaluated the number of ERCC1 nuclear foci (Figure 4A‐C) and found that ERCC1 foci formation was highest with carboplatin monotherapy. However, when carboplatin was combined with pevonedistat, there was a significant reduction in ERCC1 nuclear foci formation (Figure 4C). We tested to see the degree of double strand breaks (DSBs) by quantifying the degree of γH2AX foci formation (Figure 4A‐C), which is a known marker of DSBs.^40^ γH2AX foci formation was highest with carboplatin and did not decrease with the addition of pevonedistat (Figure 4C). Additionally, colocalisation between ERCC1 foci and γH2AX was assessed. Colocalisation between the two proteins was highest in the carboplatin treated cells, and reduced in cells treated with pevonedistat and carboplatin, suggesting that ERCC1 foci were no longer forming at areas of DNA damage when cells were exposed to pevonedistat (Figure 4D). These data support the hypothesis that, while pevonedistat treatment does not reduce the protein levels of ERCC1, it does impede the ability of cells to form ERCC1 nuclear foci in response to DNA damage.

### 3.4 Pevonedistat acts synergistically with cytotoxic chemotherapy in vivo

**FIGURE 5:**
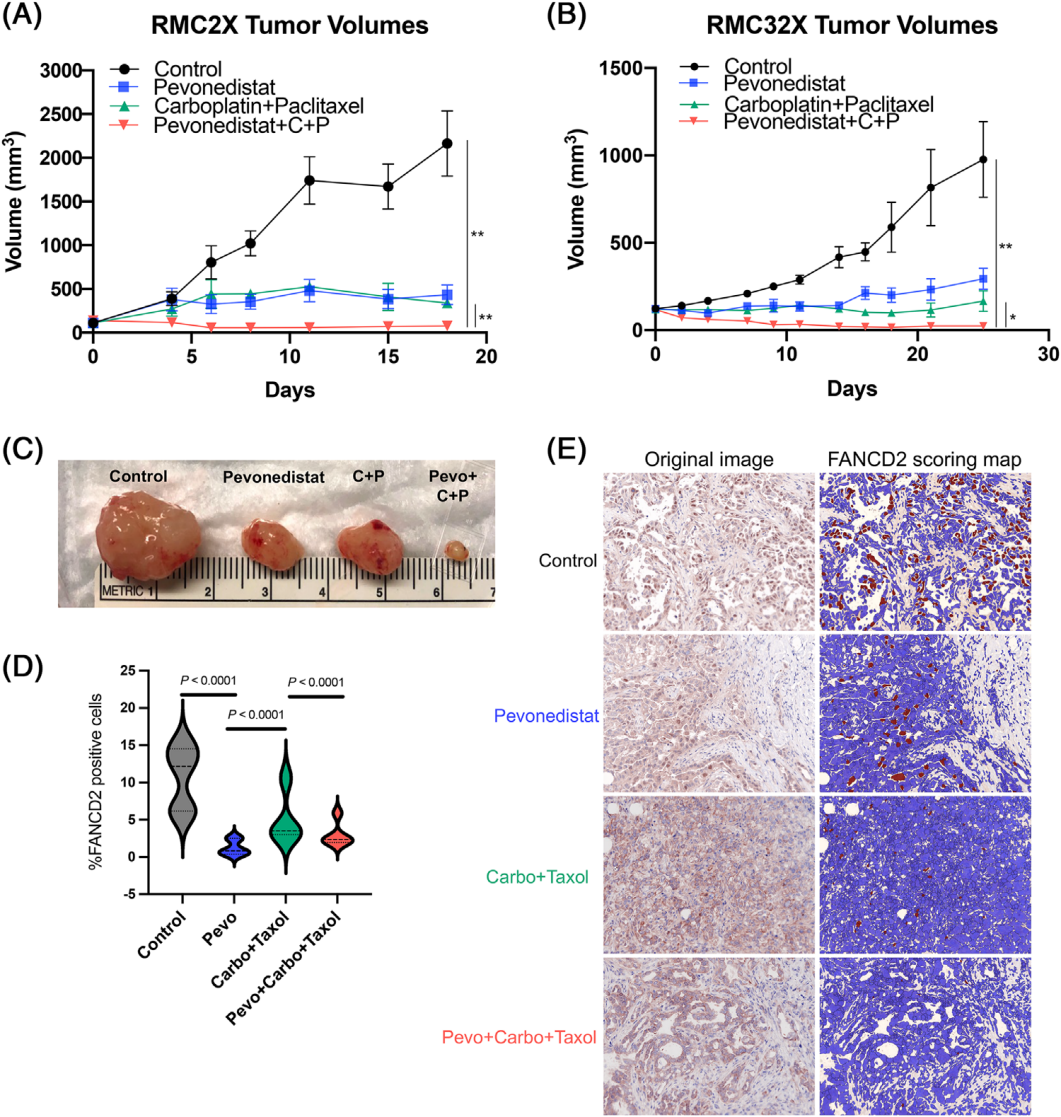
Pevonedistat acts synergistically with carboplatin and paclitaxel to inhibit renal medullary carcinoma (RMC) patient‐derived xenograft (PDX) tumour growth. The RMC2X PDX was derived from nephrectomy specimen from patient who had no prior systemic therapy, whereas RMC32X was derived from nephrectomy specimen from a patient who had received prior platinum therapy. After tumour implantation, tumours were allowed to grow to ∼200 mm^3^, at which point drug therapy would begin. Mice were treated with intraperitoneal injections of either vehicle, pevonedistat (30 mg/kg), carboplatin (80 mg/kg) with paclitaxel (20 mg/kg) or triple therapy (pevonedistat + carboplatin + paclitaxel). Each therapy arm contained five to eight mice. The vehicle and pevonedistat were given daily for 5 and 2 days off for a 7‐day cycle. Carboplatin and paclitaxel were injected once per 7‐day cycle. Tumour volumes are reported for RMC2X (A) and RMC32X (B). Triple therapy tumour volumes were compared to control tumour volumes and tumours treated with carboplatin and paclitaxel. Significant differences were evaluated with unpaired *t*‐tests. ^*^ *p* < .05, ^**^ *p* < .01. (C) Representative tumours harvested from RMC32X mice in each therapy arm. (D) Tumours from the RMC32X model were excised for immunohistochemistry staining for FANCD2 (*n* = 5 control tumours, *n* = 3 pevonedistat treated tumours, *n* = 4 carboplatin + paclitaxel treated tumours and *n* = 5 pevonedistat + carboplatin + paclitaxel treated tumours). Multiple images per tumour were taken with Vectra automated quantitative pathology imaging system and were quantified using Inform 2.5 software. Images are scored and a percent positivity is given for each image, which represents the percent of cells in a given image that are positive for DAB (i.e., FANCD2). The violin plot shows the distribution of proportions of FANCD2+ cells in each tumour. (E) Representative images of the quantitative scoring of FANCD2+ cells, which are demonstrated by the brown staining cells on the FANCD2 scoring map. Fisher's exact tests were used to evaluate differences in proportions of total FANCD2+ cells in each experimental arm.

We next tested whether pevonedistat and the clinically preferred first‐line chemotherapy combination of carboplatin + paclitaxel would effectively inhibit RMC tumour cells in vivo. Two different PDX tumour models (RMC2X and RMC32X) were used, and tumour volumes were measured after treatment with either vehicle control, pevonedistat, carboplatin + paclitaxel or pevonedistat + carboplatin + paclitaxel. In vivo experiments were ended based on the growth of the control tumours. RMC2X tumours grew faster than RMC32X tumours; thus, the experiment was ended earlier and tumours were harvested simultaneously from each experimental arm at the end of the experiment. For the RMC2X PDX, mean final tumour volumes were 2164 ± 835 mm^3^ for control, 433 ± 250 mm^3^ for pevonedistat, 339 ± 44 mm^3^ for carboplatin + paclitaxel and 75 ± 46 mm^3^ for pevonedistat + carboplatin + paclitaxel. The combination of pevonedistat with chemotherapy significantly reduced tumour volumes compared to vehicle control (*p* = .002) and chemotherapy alone (*p* = .008). For the RMC32X PDX, mean final tumour volumes at the end of treatment were 976 ± 216 mm^3^ for control, 294 ± 59 mm^3^ for pevonedistat, 166 ± 58 mm^3^ for carboplatin + paclitaxel and 24 ± 7 mm^3^ for pevonedistat + carboplatin + paclitaxel. The combination of pevonedistat with chemotherapy again significantly reduced tumour volumes compared to vehicle control (*p* = .006) and chemotherapy alone (*p* = .02). A synergistic effect was found when pevonedistat was combined with chemotherapy for both the RMC2X model (*p* < .001) and RMC32X (*p* = .002) (Figure 5A–C).

Immunohistochemistry staining for FANCD2 was performed on RMC32X tumours after excision on day 26. Pevonedistat significantly decreased the proportion of FANCD2+ cells compared to both control tumours (1.25% vs. 10.94%, *p* < .0001) and carboplatin + paclitaxel treated tumours (1.25% vs. 5.05%, *p* < .0001) (Figure 5D). The addition of pevonedistat to carboplatin and paclitaxel significantly reduced the proportion of FANCD2+ cells compared to carboplatin + paclitaxel alone (2.93% vs. 5.05%, *p* < .0001) (Figures 5D,E and S4).

## 4 DISCUSSION

The results of this study demonstrate that neddylation pathway inhibition by pevonedistat combined with carboplatin synergistically inhibits RMC both in vitro and in vivo. Phase I trial data of the triplet combination of pevonedistat with carboplatin and paclitaxel noted complete responses in patients with treatment‐refractory urothelial carcinoma and endometrial carcinoma.^22^ Our findings support the development of a phase II clinical trial of this triplet combination in patients with RMC.

Mechanistically, our results suggest that the synergy between pevonedistat and carboplatin occurs, at least partially, through the inhibition of the NER pathway. The NER pathway is a critical DNA damage repair pathway that corrects ICLs, which are the primary DNA lesions induced by platinum salts such as carboplatin. A critical component of the cellular response to ICLs is FA pathway proteins. FA proteins cooperate through a common pathway required for recognition and repair of the DNA ICLs created by carboplatin.^41^ A key node of this pathway is the FANCD2 protein, which is monoubiquitinated by the FA core complex. Monoubiquitinated FANCD2 then assembles at sites of DNA damage in the nucleus, where they colocalise with downstream effector proteins for DNA repair.^41^ Cells from patients with deficiencies in the FA proteins display abnormally high sensitivity to ICL‐inducing agents such as cisplatin and mitomycin C.^41^, ^42^ We found that RMC cells exposed to pevonedistat have a marked reduction in FANCD2 protein levels. This finding is supported by a prior study by Kee et al.,^43^ which demonstrated that pevonedistat or siRNA‐mediated inhibition of the neddylation system caused a synergistic elevation in cell sensitivity to ICL‐inducing agents across various cell lines. Specifically, pevonedistat suppressed FANCD2 foci formation at areas of DNA ICLs and significantly reduced HCT116 colon cancer cell survival in combination with cisplatin.^43^

We did not observe a synergistic effect between pevonedistat and paclitaxel in RMC cell lines. The primary mechanism of action of paclitaxel is microtubule stabilisation and is not directly involved in either proteotoxic or replication stress pathways targeted by pevonedistat. While pevonedistat only additively affected RMC cell proliferation in combination with paclitaxel, the triplet combination of pevonedistat with carboplatin and paclitaxel synergistically and potently inhibited RMC tumours.

**FIGURE 6:**
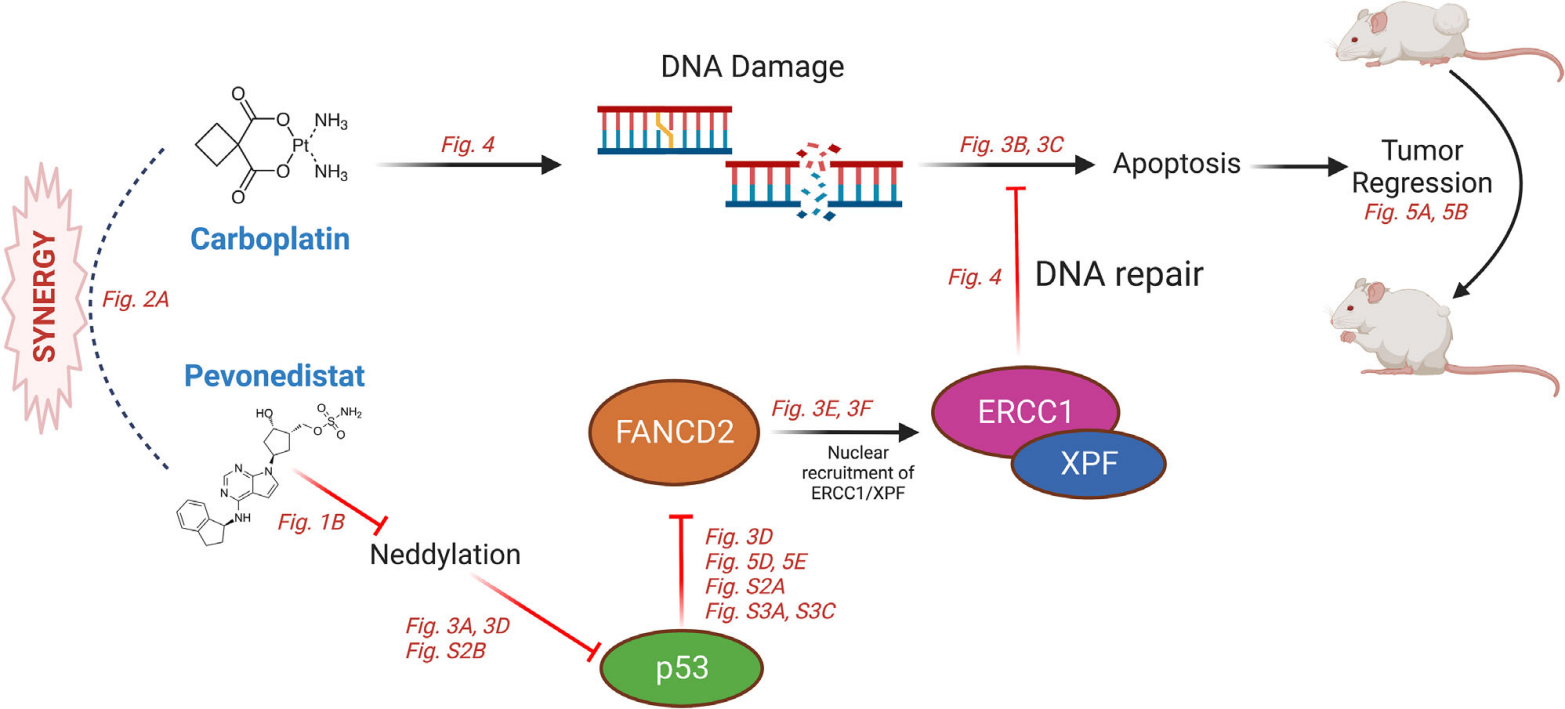
Schematic model of the synergy between carboplatin treatment and neddylation pathway inhibition by pevonedistat. Carboplatin induces DNA damage through interstrand crosslinks and subsequent double strand breaks. These are repaired through the Fanconi anaemia pathway proteins via the activation of FANCD2, which induces the nuclear recruitment of the ERCC1/XPF nucleotide excision repair complex. Pevonedistat reduces p53 neddylation, leading to FANCD2 downregulation and subsequent reduction in ERCC1 nuclear levels despite the presence of DNA damage induced by carboplatin. The inability of cells to repair this damage leads to cell death in vitro and tumour regression in vivo. Figures from this study with data demonstrating steps in this pathway are represented in red.

The exact mechanism through which pevonedistat causes a reduction in FANCD2 requires further study. We noted increased TP53 expression upon exposure to pevonedistat when compared to either control or carboplatin monotherapy. Prior studies have demonstrated that activated TP53 downregulates several FA genes including FANCD2.^35^ This downregulation decreases the ability of cells to repair DNA ICLs.^35^ Our results support the hypothesis that increased TP53 activation via the inhibition of the neddylation pathway results in FANCD2 downregulation. By knocking out p53 expression, FANCD2 expression was subsequently recovered even in the presence of pevonedistat. FANCD2 downregulation is known to attenuate the recruitment of XPF–ERCC1 endonuclease DNA damage repair complex, a critical component of the NER pathway.^21^ We accordingly found that carboplatin induces DNA damage in RMC cells as evidenced by increased γH2AX nuclear foci formation.^40^ However, pevonedistat reduces the nuclear levels of ERCC1 in the setting of carboplatin treatment. Taken together, these findings suggest that pevonedistat increases TP53 expression which reduces FANCD2 resulting in lower nuclear levels of ERCC1 and thus attenuating the ability of RMC cells to cope with DNA damage induced by platinum salts such as carboplatin (Figure 6).

While inhibition of the NER pathway is one potential mechanism of synergism between pevonedistat and carboplatin, pevonedistat has additional toxic effects that may also be particularly relevant in RMC. RMC and other SMARCB1‐deficient tumours have previously been shown to be particularly susceptible to proteotoxic stress.^6^, ^10^, ^20^ A prior study in MRT models demonstrated that pevonedistat could induce a cytotoxic response through upregulation of the unfolded protein response in SMARCB1‐deficient tumours.^20^ Given that RMC tumours are defined by loss of SMARCB1 and exhibit high levels of proteotoxic and replication stress.^8^ Inhibition of the neddylation pathway via drugs such as pevonedistat is uniquely poised to exploit both of these synthetic vulnerabilities in RMC.

RMC is an aggressive and fatal malignancy afflicting a young vulnerable population in need of new treatment options. Based on the results of our study, we demonstrate that pevonedistat combines with carboplatin synergistically in vitro and in vivo. Our mechanistic studies in vitro demonstrate that pevonedistat results in reduced FANCD2 cellular expression, possibly mediated by increased expression of TP53. Indeed, TP53 knockout in RMC2C cells restored FANCD2 expression even after exposure to pevonedistat. Our in vivo studies demonstrate that in two RMC PDX models, the addition of pevonedistat to the doublet therapy carboplatin and paclitaxel yielded a synergistic tumour shrinkage effect in RMC tumours and provides preclinical evidence to support a future clinical trial combining these agents for the treatment of RMC.

## CONFLICT OF INTEREST STATEMENT

Jose A. Karam reports honoraria for scientific advisory board memberships/consulting for Merck, Pfizer, Johnson and Johnson; stock ownership in MedTek, ROMTech; research funding to institution from Mirati, Roche/Genentech, Merck, Elypta. Pavlos Msaouel reports honoraria for scientific advisory boards membership for Mirati Therapeutics, Bristol‐Myers Squibb and Exelixis; consulting fees from Axiom Healthcare; non‐branded educational programmes supported by Exelixis and Pfizer; leadership or fiduciary roles as a Medical Steering Committee Member for the Kidney Cancer Association and a Kidney Cancer Scientific Advisory Board Member for KCCure; and research funding from Takeda, Bristol‐Myers Squibb, Mirati Therapeutics and Gateway for Cancer Research. Nizar M. Tannir reports grants/contracts from Bristol‐Myers Squibb, Nektar Therapeutics, Calithera Bioscience, Arrowhead Pharmaceuticals, Eisai and Novartis; consulting fees from Oncorena; honoraria for advisory meetings, presentations and educational events from Bristol‐Myers Squibb, Nektar Therapeutics, Exelixis, Eisai, Eli Lilly, Oncorena, Calithera, Surface Oncology, Novartis, Ipsen and Merck Sharp & Dohme; participation on a data safety monitoring board or advisory board as a member of the US RCC Advisory Board Committee for Merck; and is a stockholder of Amgen, Arcturus Therapeutics, Arcus Biosciences, Bellus Health, BioCryst, Corvus Pharmaceuticals, First Trust Amex Biotech, Johnson & Johnson, Merck, Nuvation Bio, Revolution Medicines, Spdr S&P Pharmaceuticals, Surface Oncology, Vanguard Healthcare Solutions and Xencor.

## Supporting information

Patient derived Xenografts. (A) Development of the patient derived xenograft models. Primary tumors were isolated from nephrectomy specimens. Tumor sections were then implanted subcutaneously in SCID mice and allowed to grow. Tumors that successfully grew were then serially passaged 2–3 times at which point the tumor was analyzed with STR DNA profiling to confirm the patient derived xenograft tumor was identical to the original renal tumor. PDX tumor sections were then frozen for future use. In the case of the RMC32X PDX model, the patient received chemotherapy before nephrectomy was performed. (B) Schematic of the PDX therapeutic experiments. PDX tumor fragments were thawed and implanted subcutaneously in SCID mice and allowed to grow to ∼200 mm^3^. Mice were then randomly allocated to one of four treatment arms. Treatment cycles were carried out as indicated by the figure.

Quantification of protein expression using densitometric analysis. (A–B) Western blot protein expression was quantified for FANCD2 (A) and TP53 (B) and normalized to actin levels after treatment with either control or drug therapies. Cells were exposed to vehicle control, pevonedistat 0.2 µM, carboplatin 20 µM, or pevonedistat with carboplatin for 48 hours. All samples were performed in triplicate (n = 3) using RMC2C cells and compared with unpaired t tests. Bars indicate mean and error bars indicate SD.

Quantification of protein expression after TP53 knockdown by siRNA. (A) Western blot analysis performed after 48‐hour treatment of RMC2C cells with siRNA control versus siRNA against TP53 followed by an additional 48‐hour treatment with pevonedistat (0.2 µM) plus carboplatin (20 µM) with either siRNA control vectors or siRNA against TP53. (B–C) Western blot protein expression was quantified for TP53 (B) and FANCD2 (C) in cells expressing either control or TP53 siRNA after treatment with both pevonedistat 0.2 µM and carboplatin 20 µM. All samples were performed in triplicate (n = 3) using RMC2C cells and compared with unpaired *t* tests. Bars indicate means and error bars indicate SD.

Representative immunohistochemistry images staining for FANCD2. Each row of images corresponds to a tumor section from a different treatment arm of the RMC32X treatment model. FANCD2 is stained by DAB (brown) and nuclei are stained with hematoxylin.

Supporting Information
