# Supplementary figures and images for: Neddylation inhibition sensitises renal medullary carcinoma tumours to platinum chemotherapy
Source: Clin Transl Med. 2023 May 25;13(5):e1267. doi: 10.1002/ctm2.1267 (PMC10210052; doi:10.1002/ctm2.1267)

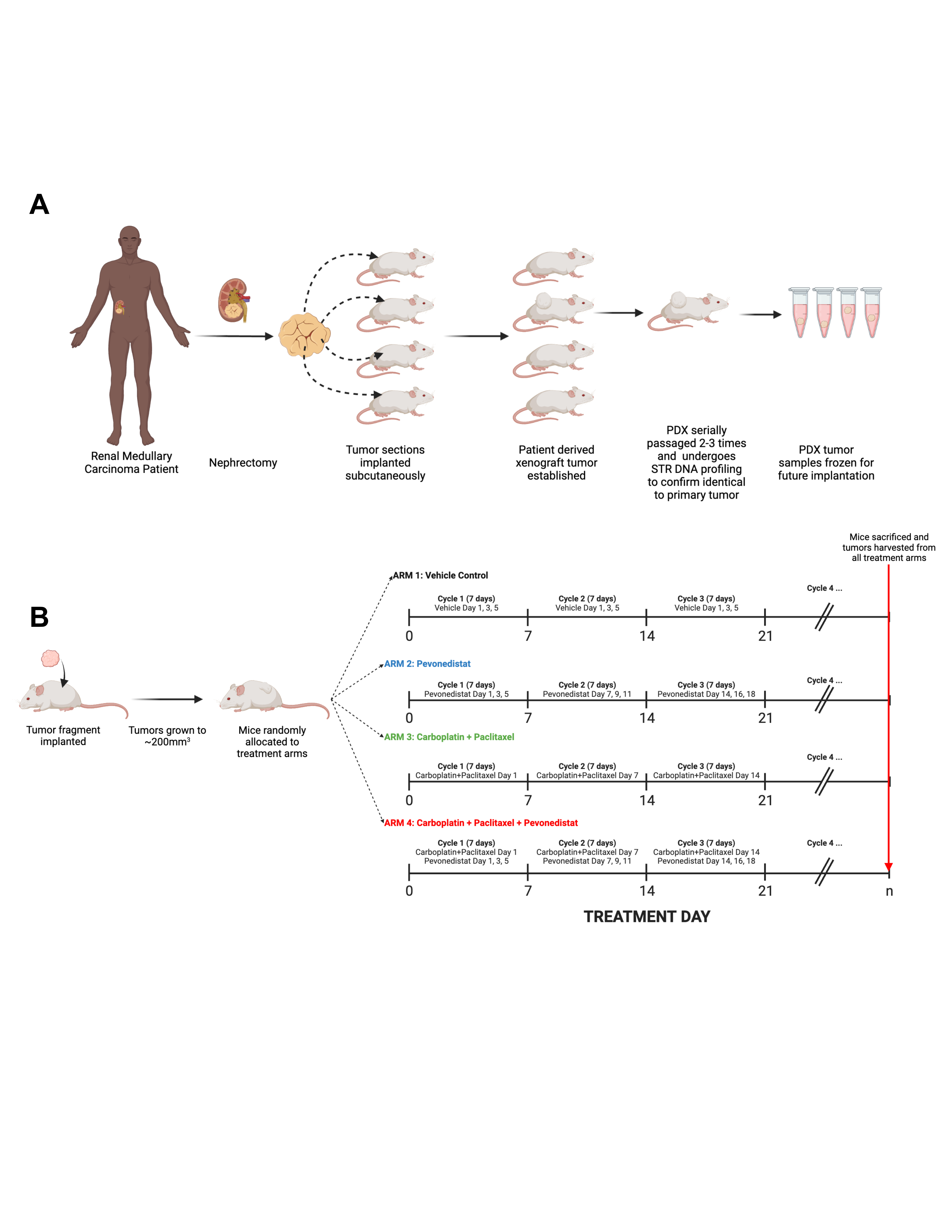

Supplement: Supplementary file 1 — Patient derived Xenografts. (A) Development of the patient derived xenograft models. Primary tumors were isolated from nephrectomy specimens. Tumor sections were then implanted subcutaneously in SCID mice and allowed to grow. Tumors that successfully grew were then serially passaged 2–3 times at which point the tumor was analyzed with STR DNA profiling to confirm the patient derived xenograft tumor was identical to the original renal tumor. PDX tumor sections were then frozen for future use. In the case of the RMC32X PDX model, the patient received chemotherapy before nephrectomy was performed. (B) Schematic of the PDX therapeutic experiments. PDX tumor fragments were thawed and implanted subcutaneously in SCID mice and allowed to grow to ∼200 mm3. Mice were then randomly allocated to one of four treatment arms. Treatment cycles were carried out as indicated by the figure. [file CTM2-13-e1267-s005.png]

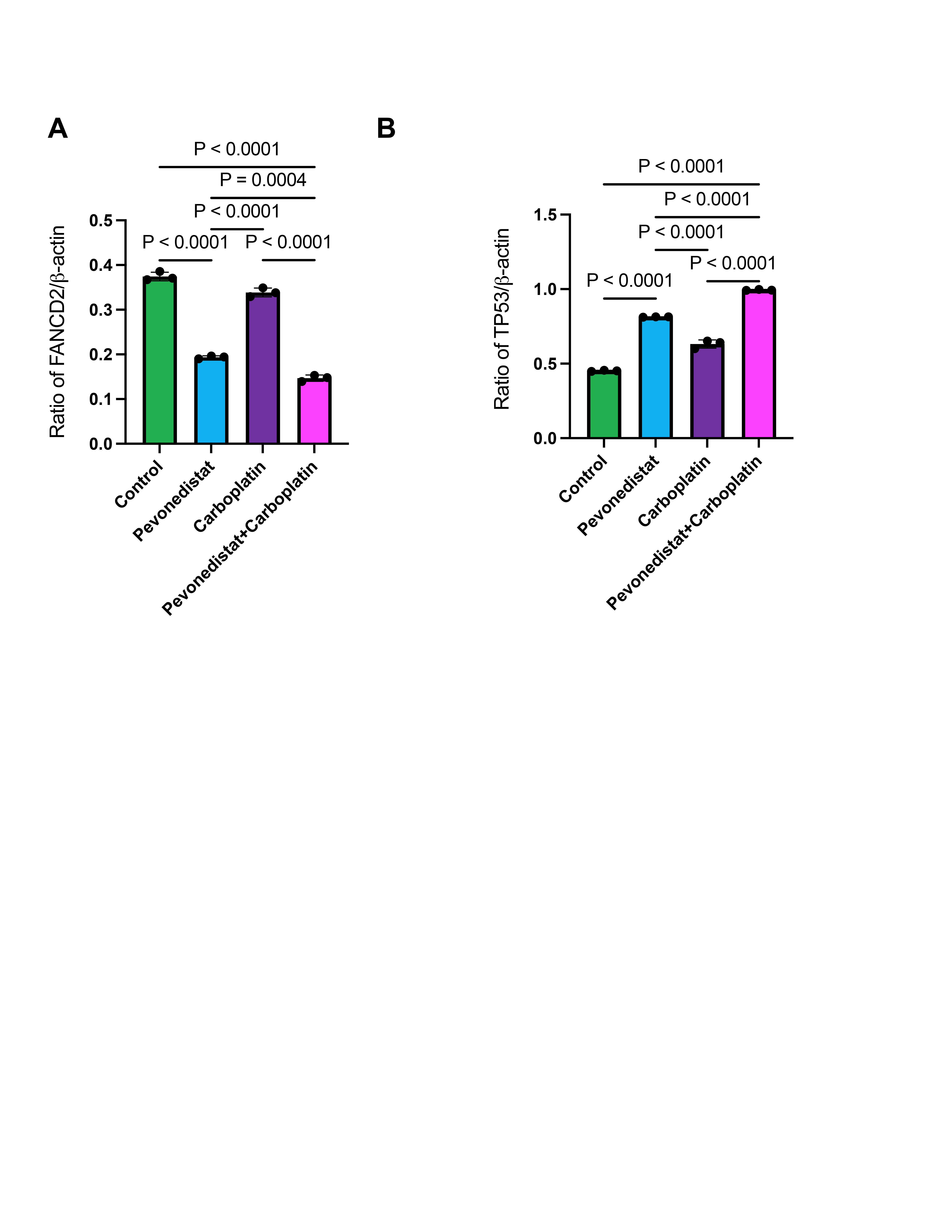

Supplement: Supplementary file 2 — Quantification of protein expression using densitometric analysis. (A–B) Western blot protein expression was quantified for FANCD2 (A) and TP53 (B) and normalized to actin levels after treatment with either control or drug therapies. Cells were exposed to vehicle control, pevonedistat 0.2 µM, carboplatin 20 µM, or pevonedistat with carboplatin for 48 hours. All samples were performed in triplicate (n = 3) using RMC2C cells and compared with unpaired t tests. Bars indicate mean and error bars indicate SD. [file CTM2-13-e1267-s003.png]

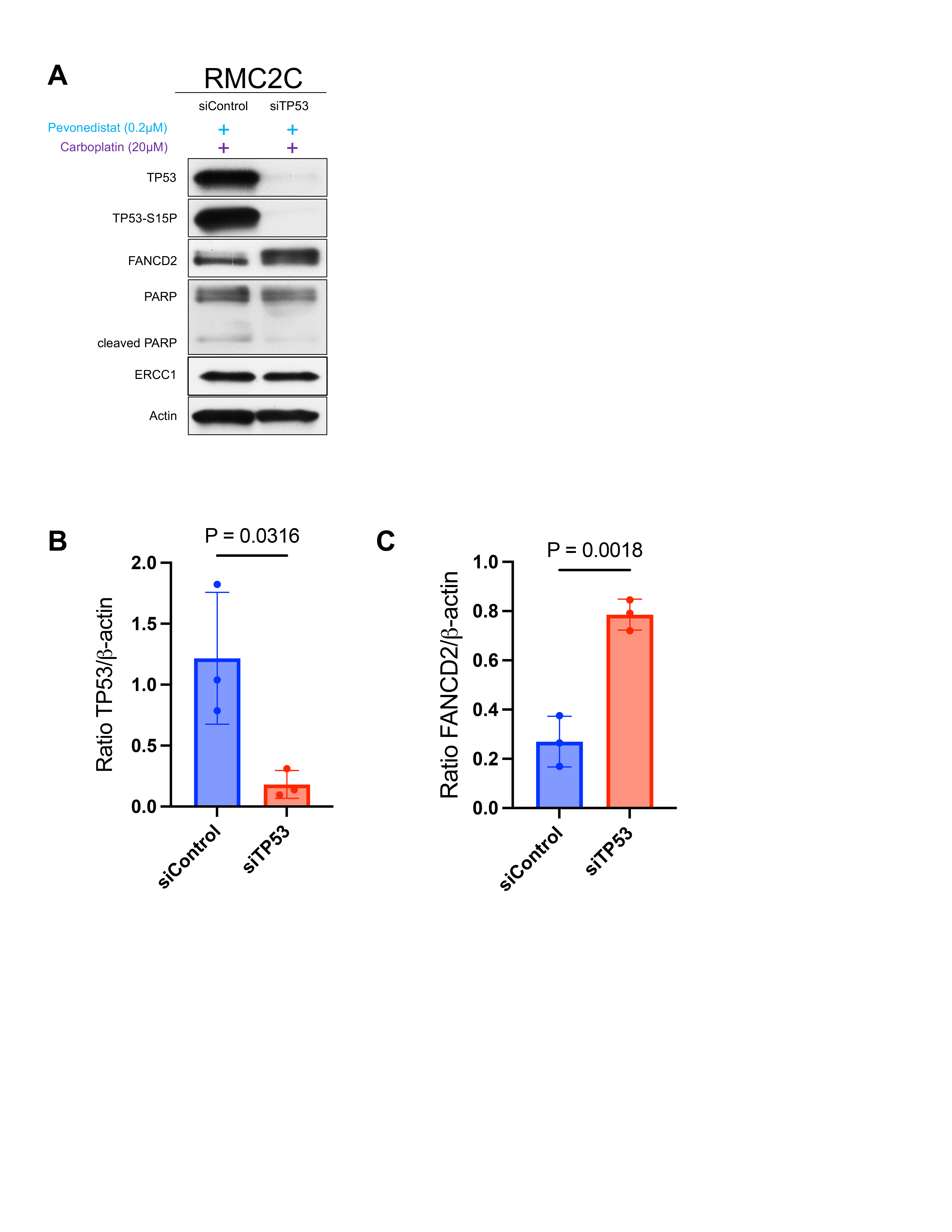

Supplement: Supplementary file 3 — Quantification of protein expression after TP53 knockdown by siRNA. (A) Western blot analysis performed after 48‐hour treatment of RMC2C cells with siRNA control versus siRNA against TP53 followed by an additional 48‐hour treatment with pevonedistat (0.2 µM) plus carboplatin (20 µM) with either siRNA control vectors or siRNA against TP53. (B–C) Western blot protein expression was quantified for TP53 (B) and FANCD2 (C) in cells expressing either control or TP53 siRNA after treatment with both pevonedistat 0.2 µM and carboplatin 20 µM. All samples were performed in triplicate (n = 3) using RMC2C cells and compared with unpaired t tests. Bars indicate means and error bars indicate SD. [file CTM2-13-e1267-s001.png]

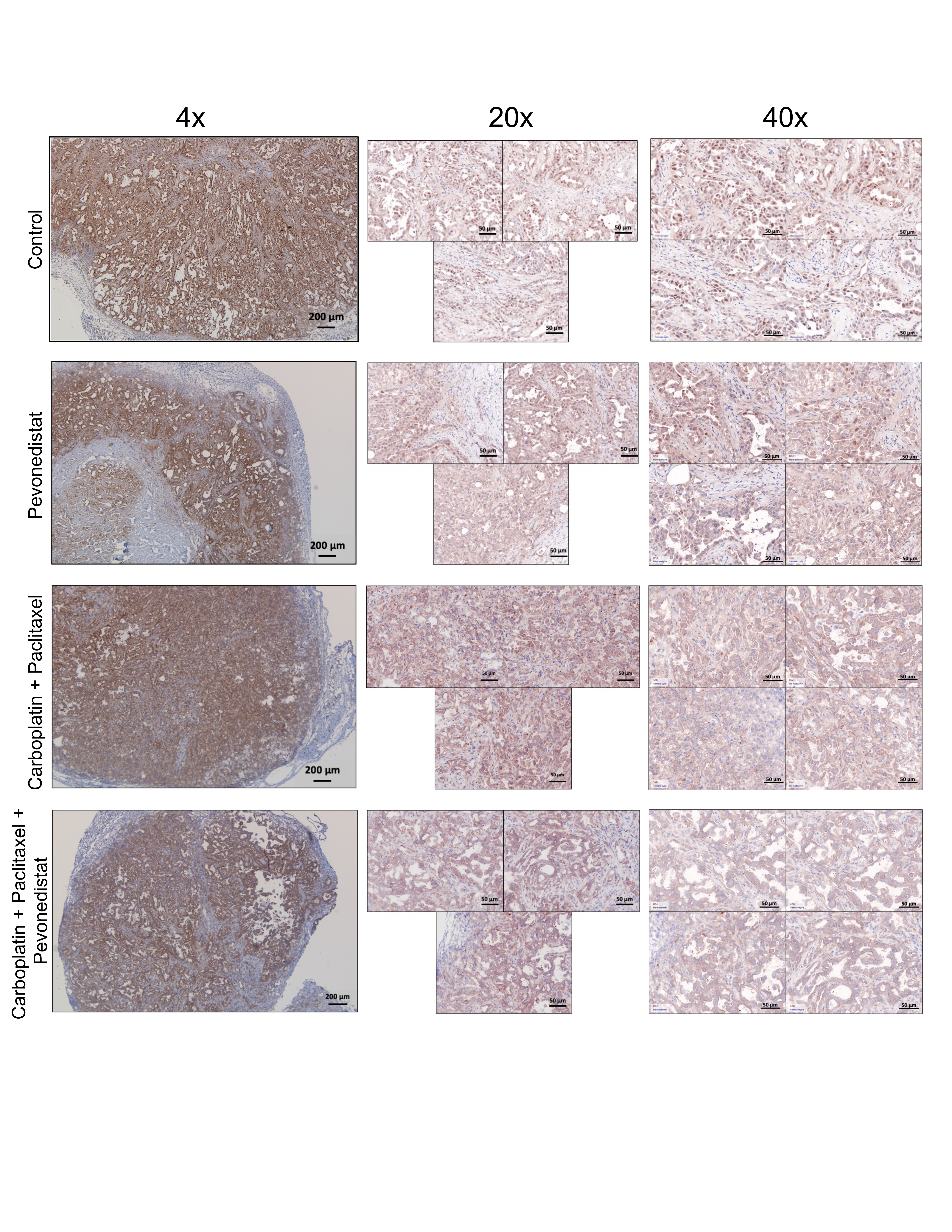

Supplement: Supplementary file 4 — Representative immunohistochemistry images staining for FANCD2. Each row of images corresponds to a tumor section from a different treatment arm of the RMC32X treatment model. FANCD2 is stained by DAB (brown) and nuclei are stained with hematoxylin. [file CTM2-13-e1267-s002.png]
